# CNT-Based Inkjet-Printed RF Gas Sensor: Modification of Substrate Properties during the Fabrication Process

**DOI:** 10.3390/s19081768

**Published:** 2019-04-13

**Authors:** Julien George, Aymen Abdelghani, Prince Bahoumina, Olivier Tantot, Dominique Baillargeat, Kamel Frigui, Stéphane Bila, Hamida Hallil, Corinne Dejous

**Affiliations:** 1 University of Limoges, CNRS, XLIM UMR 7252, F-87060 Limoges, France; aymen.abdelghani@xlim.fr (A.A.); olivier.tantot@xlim.fr (O.T.); dominique.baillargeat@xlim.fr (D.B.); kamel.frigui@xlim.fr (K.F.); stephane.bila@xlim.fr (S.B.); 2 University of Bordeaux, Bordeaux INP/CNRS, IMS UMR 5218, F-33400 Talence, France; prince.bahoumina@gmail.com (P.B.); hamida.hallil-abbas@u-bordeaux.fr (H.H.); corinne.dejous@ims-bordeaux.fr (C.D.)

**Keywords:** dielectric characterization, inkjet printing, heating influence, gas sensor, RF structure, PEDOT: PSS-MWCNT, Flexible substrate

## Abstract

This paper presents the feasibility of a fully inkjet-printed, microwave flexible gas sensor based on a resonant electromagnetic transducer in microstrip technology and the impact of the printing process that affects the characteristics of the gas sensor. The sensor is fabricated using silver ink and multi-wall carbon nanotubes (MWCNTs) embedded in poly (3,4-ethylenedioxythiophene) polystyrene (PEDOT: PSS-MWCNTs) as sensitive material for Volatile Organic Compounds (VOCs) detection. Particular attention is paid to the characterization of the printed materials and the paper substrate. The manufacturing process results in a change in relative permittivity of the paper substrate by nearly 20%. Electrical characterization, made in the presence of gas, validates our theoretical approach and the radiofrequency (RF) gas sensor proof of concept.

## 1. Introduction

Atmospheric pollution involves several particles and mixture into complex gases, such as volatile organic compounds (VOCs), where concentration is usually detected in the range of parts per million (ppm) for health and environmental applications. Most of the commercially available sensors are based on conductivity transduction, using metal oxides as a sensitive layer [1,2,3]. However, such sensors often operate at high temperatures [4]. In contrast, electromagnetic transducers can operate at room temperature [5], which is one of their advantages. To increase the sensitivity and to emphasize the selectivity, the active part is sometimes covered by a sensitive layer. Several studies have been conducted on the design of radiofrequency (RF) gas sensors in recent years and a large part of these studies converge to the use of carbon nanotubes as a sensitive element. Various studies have been carried out in recent years, showing the modification of the conductivity of carbon nanotubes and poly(3,4-ethylenedioxythiophene) polystyrene sulfonate (PEDOT: PSS) in the presence of alcohols [6,7,8,9]. Moreover, carbon nanotubes (CNTs) have interesting characteristics because of their ability to be functionalized. Several studies have been conducted with antennas and RF resonators using carbon nanotubes to detect VOCs [10,11,12]. For example, Naishadham et al. [13] have presented a sensor based on single-walled carbon nanotubes (SWCNT), functionalized with poly-aminobenzene sulfonic acid (PABS), for the detection of gas.

Printed electronics is a rapidly emerging and relatively new technology within the electronics industry [14]. It allows for the fabrication of electronic devices on flexible substrate materials, such as plastic, paper, and textiles, using electrically functional inks. The inkjet printing technique is a direct-writing technology that prints directly the structure on a substrate carrier [15,16,17]. This technology does not require a mask, or an etching technique, but uses nozzles to create droplets on demand. Our work is focused on the use of inkjet printing on flexible substrates, such as photo paper for low-cost issues or on kapton. Several studies have been performed in recent years on this subject [18,19,20]. In this paper, we have investigated the evolution of the dielectric properties due to the manufacturing process. This study explores the field of gas sensors dedicated to the detection of pollution in the air. It proposes the development of a platform for the detection, the monitoring, and the quantification of VOCs in real time. The platform uses a flexible and printed differential gas sensor based on resonant microwave transducers and polymer–carbon composite materials, such as poly (3,4-ethylenedioxythiophene) polystyrene sulfonate and multi-wall carbon nanotubes (PEDOT: PSS-MWCNTs) composite material, as sensitive layers.

The originality of this study comes from the fact that the manufacturing procedure was taken into account in the design of the sensor. In addition, we were able to correlate the variation in ethanol concentration with the variation in conductivity of the PEDOT: PSS-MWCNTs.

The paper is divided into four main parts. First, the technological choices and the operating principle of the sensor are presented. The second part deals with the fabrication process. This process requires temperature variations that implies an experimental characterization of the paper substrate properties. In the third part, the theoretical study is conducted and the design of the RF gas sensor is presented. Then, gas measurements of the sensor are exposed. Finally, before the conclusion, the sensitivity study is presented.

## 2. Technological Choices

### 2.1. Differential Microwave Resonator

In this work, our goal is to design a differential gas sensor. This design will be based on variations of the electrical properties, of the composite sensitive material, in the presence of gas. The detection principle of this sensor is based on the differential measurement of the frequency response between two identical transducers: one of reference (without a sensitive layer) and one sensitive to the presence of a gas.

**Figure 1:**
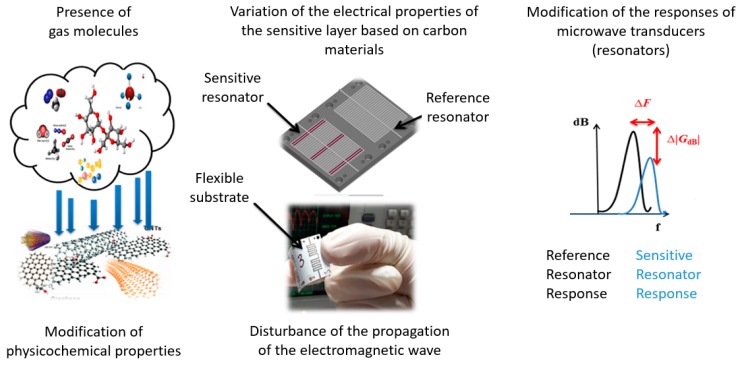
Overview of the operating principle of the differential microwave chemical gas sensor.

As shown in Figure 1, we offer a chemical gas sensor that is fully inkjet printed on a flexible paper substrate. The circuit is based on differential microwave resonance transducers that monitor the variation of the electrical properties, of the sensitive composite material, in the presence of pollutants.

In the following section, we describe the inkjet printing process we use to fabricate our test-structures.

### 2.2. Inkjet Printing Processes

To fabricate the test-structure, an inkjet printing technology process was applied by using the Dimatix Materials Printer Model DMP-2800, FUJIFILM Dimatix. This printer has 16 nozzles of 21.5 μm diameter, each separated by 254 μm [21].

In this study, the structure was directly printed on a paper substrate. Commercial conductive inks based on silver nanoparticles and on polymer-carbon composite nanomaterial (PEDOT: PSS-MWCNTs) were used as electrodes and as sensitive film, respectively. The PEDOT: PSS-MWCNTs nanomaterial was chosen because of its doping capacity for improving its conductivity. It is also a good candidate to be functionalized for increasing the sensor selectivity.

In our work, we have decided to print the sensor on a paper substrate, which is one of the most environmentally friendly materials. Based on [22,23], which demonstrate the use of paper at Ultra High Frequency (UHF) and RF frequencies, we chose Epson photo paper as substrate. Then, the inkjet printing process was applied as follows:

Step One: The structure’s fabrication starts by printing Metalon^®^ JS-B25HV silver ink as the metallic part of the sensor. That ink is produced by Novacentrix. It contains 25 wt.% of silver and its conductivity value is equal to 3.5 S·µm^−1^ by considering 2 μm of thickness.

Step Two: The device under fabrication is heated at 60 °C for 30 min in order to remove the remaining solvents and to improve the metallic part’s conductivity.

Step Three: The sensitive part of the sensor is printed with PolyInk (PEDOT: PSS-MWCNTs) containing Carbon NanoTubes (CNTs). That ink contains from 1% to 1.2% of CNTs according to the manufacturer data.

Step Four: The device is heated at 100 °C for 30 min in order to remove the remaining solvents of the printed PEDOT:PSS-WCNTs.

However, there is a wide range of papers to select from, which vary in density, coating, thickness, and dielectric properties. The electrical performances of the sensor depend on the ink and substrate properties; hence, a RF characterization of the paper substrate becomes an essential step before starting any sensor design and fabrication. Electrical characterizations of paper substrate have already been performed in [22,23] but at room temperature. In this study, we characterize the paper substrate according to the temperature cycle of the fabrication process. As described in Section three, electrical characterizations have been done in the following order: At room temperature (RT), then after a cycle at 60 °C, and finally after a cycle at 100 °C.

## 3. Characterization Method

During the last few decades, many characterization methods [24,25,26] have been developed to determine the complex properties of dielectric materials at radio frequency. For instance, we can mention the following in a non-exhaustive way: Transmission-reflection line method, open-ended coaxial probe method, free space method, and resonant method. All these methods are limited to specific operating frequencies, materials, and applications by the subject’s own constraint.

These methods are developed in time or frequency domains and are based on reflection or transmission scattering parameter measurements. Then, considering these measurements, dedicated software has been developed to extract the complex dielectric properties of the material under characterization.

**Figure 2:**
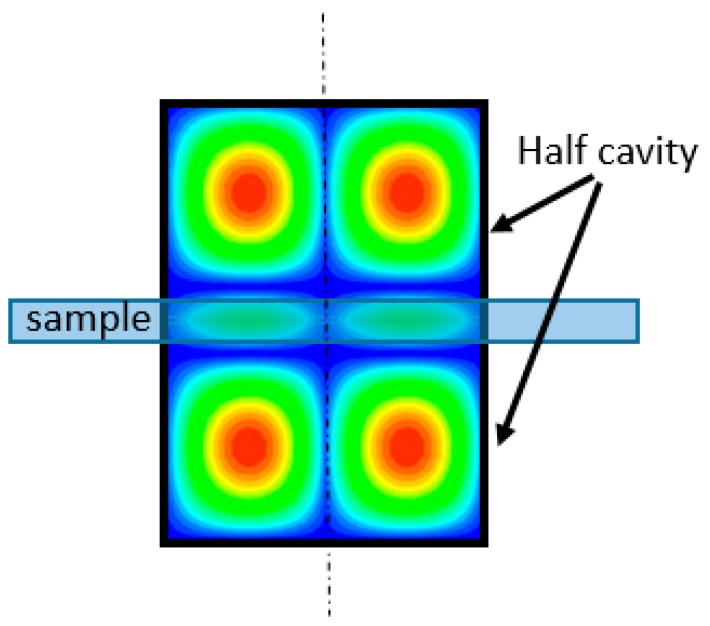
Split resonant cavity.

In our work, and in order to determine the dielectric properties of the Epson paper substrate, we used a resonant cavity method. This method consists of an empty cylindrical cavity divided into two short-circuited cylindrical waveguide sections, where the sample to be characterized is placed. The dimensions of the cavities (cavity radius, cavity height, and maximum thickness of the slot) depend on the desired characterization frequency. At last, the sample has to cover the entire cylindrical section of the cavity, as shown in Figure 2.

**Figure 3:**
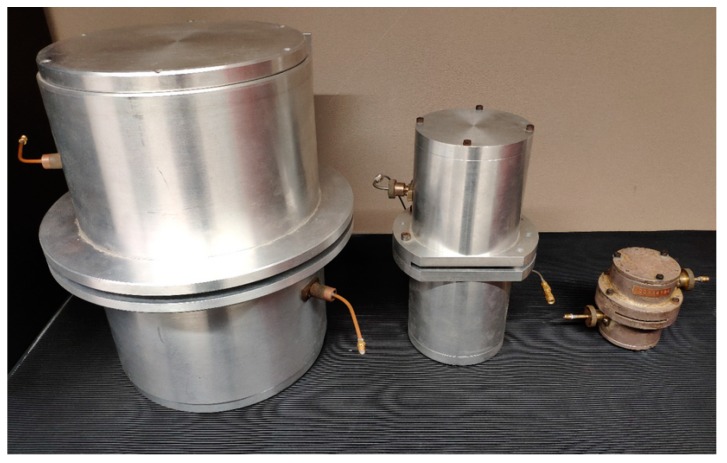
The resonant cavities used for dielectric characterization for Epson paper.

By considering both modes TE_011_ and TE_013_, the electric field that has a single azimuth component E (φ) is parallel to the plane of the dielectric sample and null on each metallic surface of the cavity. The field is concentrated in the center of the cavity that, if the slit thickness is less than 7 mm at 2.45 GHz, prevents leaks. The characterization principle consists in measuring the resonance frequencies, quality factor, and transmission parameter S_21_ (for the coupling) for the TE_011_ and TE_013_ modes. The aim is to accurately determine the dimensions and the conductivity, which depends on the temperature of the cavity. Finally, it is sufficient to re-measure these same parameters in one of the two modes, with a dielectric element to be characterized. This characterization method is presented in detail in [27,28,29]. The cavities used in this study and presented in Figure 3 have been designed by our team.

The aim of this study is to shed light on the impact of the printing cycle of our gas sensor; we characterize the substrates in the same context. First, we characterize the sample at room temperature. Then, we place the substrate in a heat chamber at 60 °C for 30 min. After two minutes, the photo paper substrate is brought back to room temperature and then measured again. This temperature corresponds to the temperature of the heating plate of the Dimatix, when printing the device. Finally, the same process is done at 100 °C. In the manufacturing process, this temperature is raised to dry out the solvent of PEDOT: PSS. To be sure that the temperature of the device to be measured is stabilized at room temperature, it is sufficient to check that the resonance frequency of the cavity does not shift over time.

The extracted dielectric properties are shown in Table 1.

According to Table 1, we observe that the relative permittivity is inversely proportional to the frequency for each temperature step in the heating process. In conclusion, this study proves that the impact of the temperature cycle of the printing process is a diminution of the relative permittivity of the photo paper substrate all along the manufacturing process of our gas sensor.

Following the characterization procedure of the paper substrate, we proceed to the design of the gas sensor according to several constraints. Hereafter, the design procedure is described.

## 4. Theoretical Study

### 4.1. Design of the Gas Sensor

The frequency range of the gas control test setup is limited from 2 to 6 GHz. These frequency test constraints also imply the dimensions of the test cell in which the sensor is integrated. Our gas sensor was designed according to all these constraints (operating frequency and dimensions).

**Figure 4:**
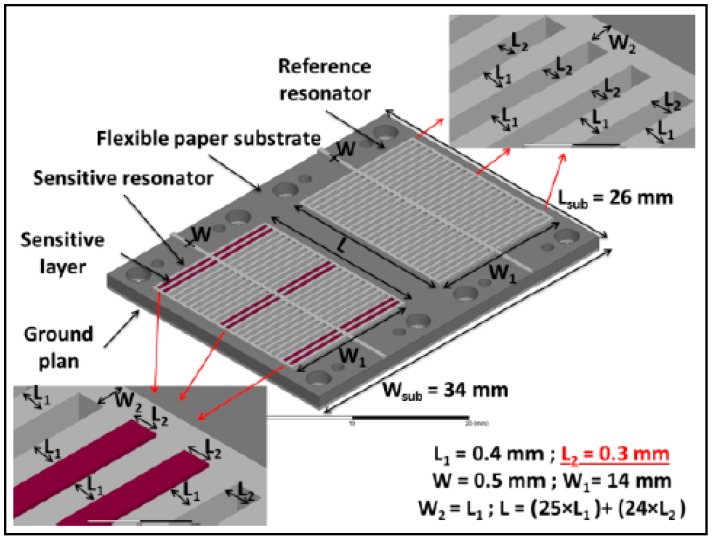
Design of the gas sensor.

The structure is composed of two identical resonators [30]. Each resonator consists of two parallel networks of 50 electrodes. The gap (L2) between two successive electrodes is equal to 300 μm. The width of each resonator (W1) is 14 mm and its main length (L) is 17.2 mm. Microstrip lines are considered as Input—Output ports in order to offer significant advantages on flexibility in the design and in the measurements of the gas sensor. To obtain an impedance matching of 50 Ω, considering the paper substrate properties, the width of each access line (W) is 0.5 mm. The geometry of the whole structure is presented in Figure 4.

Keep in mind, the structure with two resonators permits a differential detection to be obtained. Indeed, a resonator without a sensitive layer is considered as the reference channel, while the other resonator is the sensitive channel functionalized with a sensitive material.

### 4.2. Electromagnetic Simulations

The Ansys HFSS 3-D full-wave electromagnetic field simulator was used for designing the gas sensor described previously.

**Figure 5:**
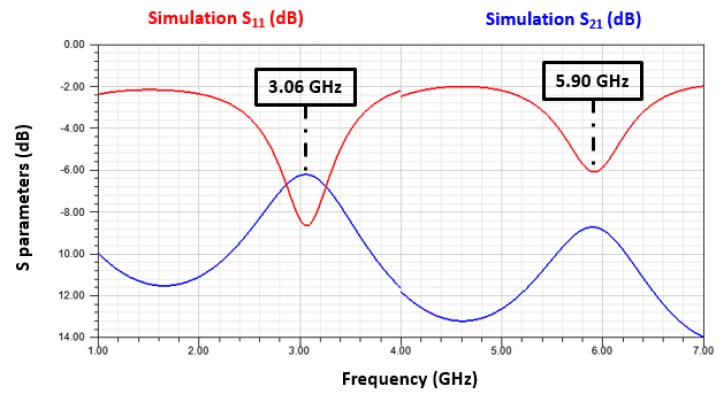
Simulation of the gas sensor’s electrical behavior. (Reference resonator).

All of the dimensions of the resonator were simulated to optimize its electrical behavior in the operating frequency range from 1 to 6 GHz. We consider the two first resonant modes of the resonator. The results of the resonator without a sensitive layer are illustrated in Figure 5.

We observe that the resonant frequencies of the first and second modes of the reference resonator are respectively 3.06 and 5.90 GHz.

**Figure 6:**
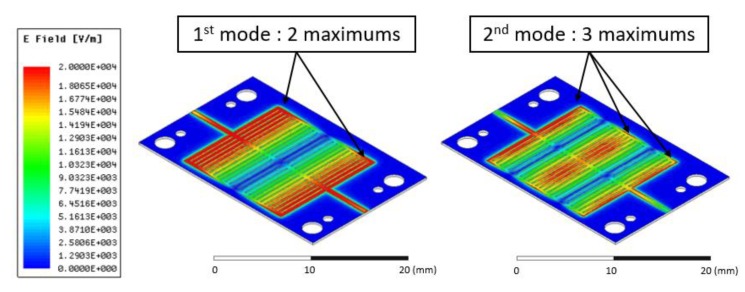
Magnitude of electrical fields in the structure.

In order to maximize the interaction with the sensitive layers, electromagnetic fields have been calculated. Figure 6 presents the distribution of the E fields for the first and second resonances, where we observe that:

- For the 1st mode: The maximum E-fields are located at the input and output of the structure.
- For the 2nd mode: The maximum E-fields are located at the input, output, and in the middle of the structure.

After studying the reference resonator, we then considered the resonator with sensitive material. As mentioned previously, this material is PEDOT: PSS-MWCNTs.

These layers are deposited in 300-μm-wide gaps, relative to the three zones where the E field is the maximum for both modes. We seek here to favor the interactions with the electric (E) field and thus to create a capacitive disturbance effect. Using electromagnetic simulations, we optimized the number of PEDOT: PSS-MWCNTs layers in order to obtain a significant electrical effect, while maintaining the accuracy of the fabrication process. Under these conditions, the number of layers we considered is equal to five, with a total thickness of 2 μm. A conductivity equal to 50 × 10^3^ S·m^−1^ is obtained for these five layers. This value was deduced from electromagnetic retro-simulation based on the Ansys HFSS.

**Figure 7:**
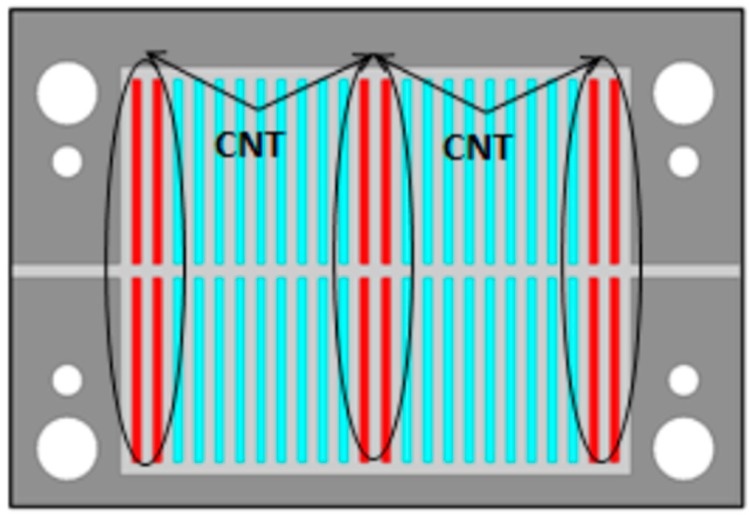
Location of the sensitive layer: 1st and 2nd mode.

Figure 7 presents the location of the sensitive pattern optimized by simulation for the five deposited layers of sensitive ink. Here, we consider the first and second resonant mode.

**Figure 8:**
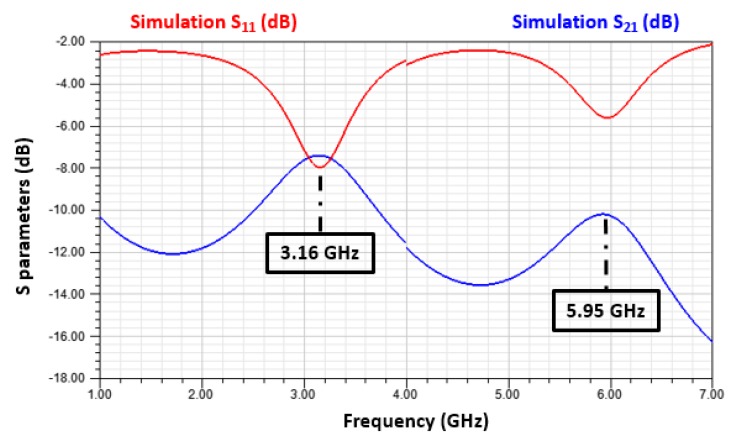
Simulation of the gas sensor’s electrical behavior. (Sensitive resonator with PEDOT: PSS-MWCNTs).

The simulation results of the device with sensitive layers are illustrated in Figure 8.

The resonant frequencies of the first and second modes of the sensitive resonator are respectively equal to 3.16 and 5.95 GHz. Printing sensitive layers, as presented in Figure 7, allows us to observe a shift of 10 MHz for the first mode and 5 MHz for the second one. In conclusion, because the first mode is more sensitive to the presence of the PEDOT: PSS-MWCNTs layers, we only consider the first mode for the rest of the study.

## 5. Experimental Study and Discussion

**Figure 9:**
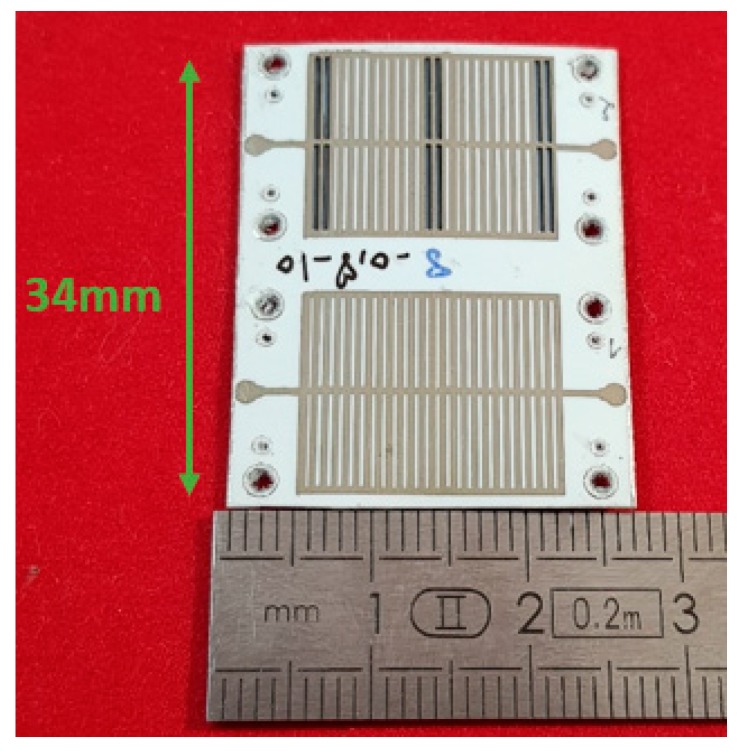
Illustration of the structure with the sensitive layers located at the maximum electric (E) fields of both modes.

The structures were printed by using the Dimatix inkjet printer (2800 series) with a silver thickness of 2 μm on the paper substrate. Figure 9 shows the two manufactured resonators of the gas sensor without and with five layers of PEDOT: PSS-MWCNTs, as indicated previously. A ground plane in aluminum was glued on the bottom face of the substrate.

After the fabrication step, the gas sensor was characterized by measuring the scattering (S) parameters without, then with, the presence of gas.

### 5.1. Measurements without Gas

**Figure 10:**
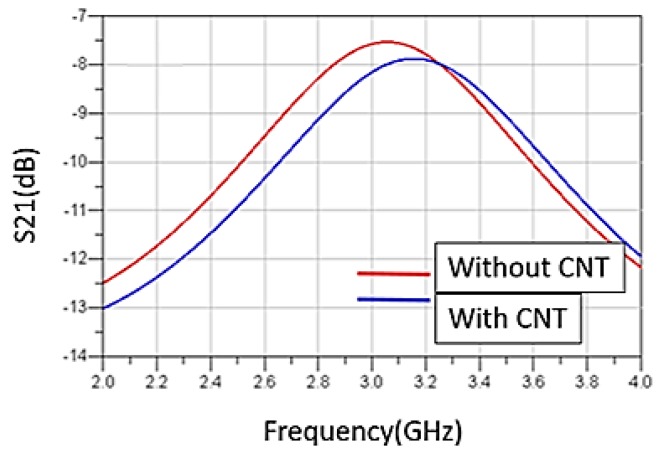
Measurements of the first mode without PEDOT: PSS-MWCNTs (red) and with it (blue).

Electrical characterizations were performed over the frequency range from 2 to 4 GHz with the MS2026B Vector Network Analyzer (VNA), calibrated for 4001 points. Figure 10 shows the experimental S parameters at room temperature for the resonators with and without sensitive layers. The resonators operate on their first resonant mode.

A frequency shift appears between the two cases. This shift is due to the presence of the PEDOT: PSS-MWCNTs. It has to be considered for defining the sensitivity of the RF gas sensor.

The resonance frequencies of the reference (FrS21r) and of the sensitive (Fr21s) resonators are given in Table 2. The good correlation between the experimental and simulation results validates our EM model and design. Furthermore, that validates the characterization of the dielectric properties of the paper substrate described previously.

### 5.2. Measurements under Gas

**Figure 11:**
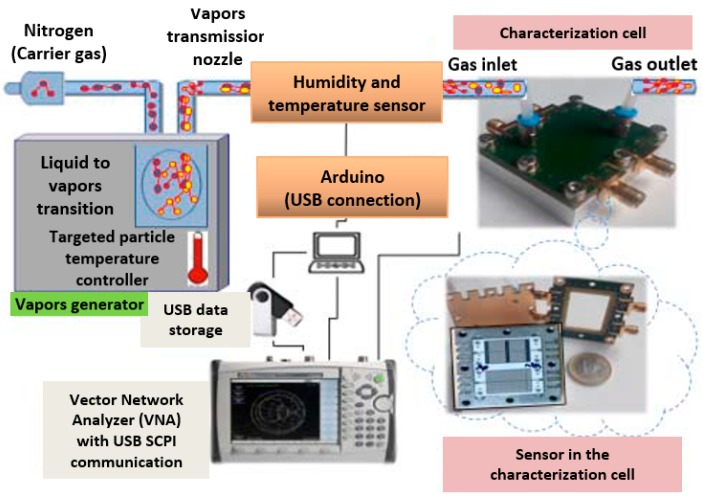
The measuring bench under gas.

The experimental configuration, summarized in Figure 11, for the detection of volatile organic compounds (VOCs) has already been described previously in [31]. It is based on a vapor generator (CALIBRAGE PUL 110), which is used to generate and control the concentration of targeted particles from a liquid in a permeation bottle or tube heated at constant temperature (60 °C for liquid ethanol and 80 °C for liquid toluene). These vapors are transported by nitrogen as carrier gas at a constant flow rate equal to 0.112 L/min [32]. According to the capabilities, characteristics, and performances of the vapor generator, we have defined a typical sequence of concentrations (C) of targeted particles vapor for which the pressure is almost constant. Thus, the detection of the vapors of ethanol and of toluene as targeted volatile organic compounds were carried out with the following concentration sequence: 0, 500, 0, 500, 0, 1000, 0, 1000, 0, and 1300 ppm. Each concentration step lasted 10 min, and the sequence was started after a first rinsing step under nitrogen for 450 min to ensure stability at room temperature. The 0 ppm corresponds to the exposure of nitrogen only. Since the permittivity of ethanol vapor is different from that of nitrogen, for the relevant frequencies, there is a change in the effective permittivity of the sensor environment and in the electrical properties of the sensitive layer [33]. All detections were carried out under the same conditions, which is to say at a fixed ambient temperature equal to 26 °C and a relative humidity (RH) of 32%.

Measurements of the resonance frequencies of the device as a function of the concentration of ethanol vapor are presented in Table 3. We note that the frequencies decrease with the increase of ethanol concentration. Moreover, we observe that the reference also varies in the presence of gas. The differential detection is necessary to detect only the variation related to the sensitive layers. To calculate the sensitivity of the sensor, we use the following formula:

$$\left(sensitivity=\frac{\Delta F\_S21}{C\left(ppm\right)}\right)$$

ΔF_S21: Differential frequency for the same structure before and after being under gas; C(ppm): Gas concentration. 

According to Table 3, the sensitivity of the reference resonator is equal to −1.23 kHz/ppm and to −4.6 kHz/ppm for the sensitive resonator. The RF gas sensor sensitivity is equal to the difference between the reference and the sensitive resonators for a specific gas concentration. This approach allows us to consider only the sensitivity due to PEDOT: PSS-MWCNTs. Finally, the gas sensor sensitivity is equal to −3.37 kHz/ppm.

## 6. Sensitivity Study

To estimate the behavior of the resonator in the presence of gas, simulations were performed by varying the value of the conductivity of the sensitive material. The objective here is to quantify the variation of the conductivity of the sensitive material, according to the variations of the gas concentration. From the measurements presented in Table 3, we find that a variation of the concentration from 0 ppm to 1300 ppm generates a variation of 6 MHz. Based on the EM model of the sensitive resonator validated previously, we performed simulations of the structure by varying the conductivity of the sensitive material. Our objective is to obtain a frequency shift of 6 MHz by EM simulation.

In Table 4, we present the simulation results of the resonance frequency variation of the sensitive channel versus the variation of conductivity from 0% to 30%.

**Figure 12:**
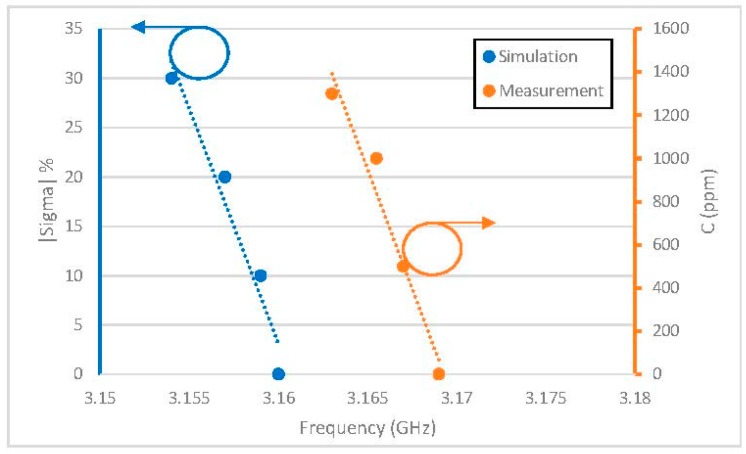
Comparison between measurement and simulation.

Figure 12 shows the resonance frequency of the sensor element as a function of the conductivity variation on the left for the simulation (blue curve) and the gas concentration on the right (orange curve) for the measurement. The same behavior can be observed between measurement and simulation. Thus, there is a linear relationship between gas concentration and conductivity of the PEDOT: PSS-MWCNTs. To obtain a frequency shift of 6 MHz (as in the experimental), we need to consider a conductivity variation equal to 30%. That means that, for a concentration of 1300 ppm, the conductivity of the sensitive material varies by 30% due to the effect of the gas.

## 7. Conclusions

In this article, the proof-of-concept of a RF gas (VOCs) sensor is experimentally demonstrated. The sensor is composed of silver ink as conductive material and PEDOT: PSS-MWCNTs as sensitive material.

One of the objectives of this paper was to understand how the fabrication process influences the electrical behavior of the resonator-based gas sensor. We used a classical inkjet printing process for printing the test structures on a paper substrate. Due to the fabrication process, we have demonstrated that the dielectric properties of the paper substrate are modified (compared to the properties given by the manufacturer). As explained in the article, the paper’s characterization was done after several temperature cycles imposed by the fabrication process. At 2.45 GHz, a variation of the relative permittivity equal to 19% was measured from the initial value. This large variation was then considered during the design of the gas sensor, along with the electrical conductivities of the printed materials. The latter were additionally characterized during this study.

The second objective of this study was to design a differential gas sensor, based on variations of the electrical properties of the sensitive composite material in the presence of gas, in the microwave frequency range from 1 to 6 GHz. The detection principle of this sensor is based on the differential measurement of the frequency response of two identical transducers: one of reference (without sensitive layer) and one sensitive to the presence of a gas. 

The last objective was to validate the theoretical approach by measurements. Thus, ethanol vapor concentrations were measured in a real time detection procedure. A sensitivity (shift in frequency versus gas concentration) of the gas sensor equal to −3.37 KHz/ppm was estimated. This result is encouraging and demonstrated the proof-of-concept of our gas sensor.

Finally, based on retro-simulation, we have established a model containing the variation of the conductivity of sensitive material versus gas concentration. This model is useful for understanding the causes of the frequency shift of the sensor in presence of gas. This model will be used for the design of future sensors.

## Figures and Tables

**Table 1:** Evolution of electrical properties.

	At Room Temperature (RT)		After 60 °C Cycle		After 100 °C Cycle	
Frequency (GHz)	$\varepsilon_{r}$	tgδ	$\varepsilon_{r}$	tgδ	$\varepsilon_{r}$	tgδ
2.45	3.3	0.12	3.24	0.117	2.66	0.0364
4.7	3.1	0.09	3.01	0.1	2.66	0.0045
10	2.86	0.017	2.8	0.094	2.57	0.0038

**Table 2:** The difference between simulation and measurement.

SIMULATION		MEASUREMENT	
FrS21r (GHz)	FrS21s (GHz)	FrS21r (GHz)	FrS21s (GHz)
3.06	3.16	3.06	3.169

**Table 3:** Frequency shift versus ethanol concentration.

C(ppm)	FrS21r (GHz)	FrS21s (GHz)
0	3.06	3.169
500	3.0595	3.167
1000	3.059	3.1655
1300	3.0584	3.163

**Table 4:** Variation of the conductivity as a function of the resonance frequency.

Conductivity Variation	FrS21s (GHz)
−30%	3.154
−20%	3.157
−10%	3.159
0%	3.16

## References

[1] Courbat J., Briand D., Yue L., Raible S., De Rooij N.F. Ultra-Low power metal-oxide gas sensor on plastic foil. Proceedings of the IEEETRANSDUCERS 2009—2009 International Solid-State Sensors, Actuators and Microsystems Conference.

[2] Dey A. (2018). Semiconductor metal oxide gas sensors. Mater. Sci. Eng. B.

[3] Courbat J., Briand D., Yue L., Raible S., De Rooij N.F. (2012). Drop-coated metal-oxide gas sensor on polyimide foil with reduced power consumption for wireless applications. Sens. Actuators B.

[4] Rieu M., Camara M., Tournier G., Viricelle J.P., Pijolat C., De Rooij N.F., Briand D. (2016). Fully inkjet printed SnO_2_ gas sensor on plastic substrate. Sens. Actuators B Chem..

[5] Hartwig M., Zichner R., Joseph Y. (2018). Inkjet-Printed Wireless Chemiresistive Sensors—A Review. Chemosensors.

[6] Xia Y., Ouyang J. (2011). PEDOT:PSS films with significantly enhanced conductivities induced by preferential solvation with cosolvents and their application in polymer photovoltaic cells. J. Mater. Chem..

[7] Alemu D., Wei H.-Y., Ho K.-C., Chu C.-W. (2012). Highly conductive PEDOT:PSS electrode by simple film treatment with methanol for ITO-free polymer solar cells. Energy Environ. Sci..

[8] Mahdavian L., Monajjemi M., Mangkorntong N. (2009). Sensor Response to Alcohol and Chemical Mechanism of Carbon Nanotube Gas Sensors, Fullerenes. Nanotub. Carbon Nanostruct..

[9] Sinha N., Ma J., Yeow J.T.W. (2006). Carbon nanotube-Based Sensors. J. Nanosci. Nanotechnol..

[10] Shen S., Fan Z., Deng J., Guo X., Zhang L., Liu G., Tan Q., Xiong J. (2018). An LC Passive Wireless Gas Sensor Based on PANI/CNT Composite. Sensors.

[11] Bahoumina P., Hallil H., Lachaud J.L., Abdelghani A., Frigui K., Bila S., Baillargeat D., Ravichandran A., Coquet P., Paragua C. (2017). Microwave flexible gas sensor based on polymer multi wall carbon nanotubes sensitive layer. Sens. Actuators B Chem..

[12] Chopra S., Natarajan S., Rao A.M. Gas sensing using Carbon nanotube-based resonator. Proceedings of the Sensors, 2004 IEEE.

[13] Naishadham K., Tentzeris M.M., Shaker H., Lee G. Novel highly-sensitive antenna-based “smart skin” gas sensor utilizing carbon nanotubes and inkjet printing. Proceedings of the IEEE International Symposium on Antennas and Propagation (APSURSI).

[14] Huang Q., Zhu Y. (2019). Printing Conductive nanomaterials for flexible and Stretchable Electronics: A Review of Materials, Processes, and Applications. Adv. Mater. Technol..

[15] Huang Q., Al-Milaji K.N., Zhao H. (2018). Inkjet Printing of Silver Nanowires for Stretchable Heaters. ACS Appl. Nano Mater..

[16] Cui Z., Han Y., Huang Q., Donq J., Zhu Y. (2018). Electrohydrodynamic printing of silver nanowires for flexible and stretchable electronics. Nanoscale.

[17] Tavakoli M., Malakooti M.H., Paisana H., Ohm Y., Green Marques D., Alhais Lopes P., Piedade A.P., De Almeida A.T., Majidi C. (2018). EGaIn-Assisted Room-Temperature Sintering of Silver Nanoparticles for Stretchable, Inkjet-Printed, Thin-Film Electronics. Adv. Mater..

[18] Tahir F.A., Shamim A., Cheema H.M., Ahmed S. (2015). Compact Kapton-based Inkjet Printed Multiband Antenna for Flexible Wireless Devices. IEEE Antennas Wirel. Propag. Lett..

[19] Heirons J., Sanz-Izquierdo B., Jun S. Inkjet Printed Dual Band Antenna for Paper UAVs. Proceedings of the 11th European Conference on Antennas and Propagation.

[20] Dujardin E., Ebbesen T.W., Yianilos P.N., Treacy J.M.M., Krishnan A. (1998). Young’s modulus of single-walled nanotubes. Phys. Rev. B.

[21] (2008). DMP-2800 Series Printer & DMC-11600 Series Cartridge FAQs.

[22] Rida A., Vyas R., Tentzeris M.M., Yang L. (2007). RFID Tag and RF Structures on a Paper Substrate Using Inkjet-Printing Technology. IEEE Trans. Microw. Theory Tech..

[23] Yang L., Tentzeris M.M. 3D Multilayer Integration and Pack-aging on Organic/Paper Low-Cost Substrates for RF and Wireless Appli-cations. Proceedings of the ISSSE ‘07 International Symposium on Signals, Systems and Electronics.

[24] Guillon P., Garault Y. (1981). Complex Permittivity of MIC Substrate. AEU Inter. J. Electron. Commun..

[25] Janezic M.D., Baker-Jarvis J. (1999). Full-wave analysis of a split-cylinder resonator for nondestructive permittivity measurements. IEEE Trans. Microw. Theory Tech..

[26] Dankov P.I., Hadjistamov B.N. Characterization of microwave substrates with split-cylinder and split-coaxial-cylinder resonators. Proceedings of the 2007 European Microwave Conference (EuMC).

[27] Derby B. (2010). Inkjet Printing of Functional and Structural Materials: Fluid Property Requirements, Feature Stability, and Resolution. Annu. Rev. Mater. Res..

[28] Tantot O., Passerieux D., Delhote N., Verdeyme S., Rammal J. Monitoring of electromagnetic characteristics of split cylinder resonator and dielectric material for temperature characterization. Proceedings of the 2014 44th European Microwave Conference (EuMC).

[29] Di Marco D.D., Drissi K., Delhote N., Tantot O., Geffroy P.-M., Verdeyme S., Chartier T. (2016). Dielectric properties of pure Alumina from 8 GHz to 73 GHz. J. Eur. Ceram. Soc..

[30] Bahoumina P., Hallil H., Lachaud J.L., Rebière D., Dejous C., Abdelghani A., Frigui K., Bila S., Baillargeat D., Zhang Q. Chemical gas sensor based on novel capacitive microwave flexible transducer and composite polymer carbon nanomaterials. Proceedings of the 2017 Symposium on Design, Test, Integration and Packaging of MEMS/MOEMS (DTIP).

[31] Sheikhi M.H., Pourfath M., Moradi M., Asad M. (2015). High sensitive and selective flexible H 2 S gas sensors based on Cu nanoparticle decorated SWCNTs. Sens. Actuators B Chem..

[32] Nikolaou I., Hallil H., Conédéra V., Deligeorgi G., Dejous C., Rebière D. (2016). Inkjet-printed graphene oxide thin layers on love wave devices for humidity and vapor detection. IEEE Sens. J..

[33] Ibrahim D.S., Mohd Razib M.A., Jarin S., Tomal A.N.M., Rana M. (2017). Review on Recent Advances of CNTs as Gas Sensors. Sens. Rev..

